# The Effects of Extra-Somatic Weapons on the Evolution of Human Cooperation towards Non-Kin

**DOI:** 10.1371/journal.pone.0095742

**Published:** 2014-05-05

**Authors:** Tim Phillips, Jiawei Li, Graham Kendall

**Affiliations:** 1 Independent Researcher, Birmingham, United Kingdom; 2 Automated Scheduling, Optimisation and Planning (ASAP) research group, School of Computer Science, University of Nottingham, Nottingham, United Kingdom; 3 University of Nottingham Malaysia Campus, Broga, Malaysia; Durham University, United Kingdom

## Abstract

Human cooperation and altruism towards non-kin is a major evolutionary puzzle, as is ‘strong reciprocity’ where no present or future rewards accrue to the co-operator/altruist. Here, we test the hypothesis that the development of extra-somatic weapons could have influenced the evolution of human cooperative behaviour, thus providing a new explanation for these two puzzles. Widespread weapons use could have made disputes within hominin groups far more lethal and also equalized power between individuals. In such a cultural niche non-cooperators might well have become involved in such lethal disputes at a higher frequency than cooperators, thereby increasing the relative fitness of genes associated with cooperative behaviour. We employ two versions of the evolutionary Iterated Prisoner's Dilemma (IPD) model – one where weapons use is simulated and one where it is not. We then measured the performance of 25 IPD strategies to evaluate the effects of weapons use on them. We found that cooperative strategies performed significantly better, and non-cooperative strategies significantly worse, under simulated weapons use. Importantly, the performance of an ‘Always Cooperate’ IPD strategy, equivalent to that of ‘strong reciprocity’, improved significantly more than that of all other cooperative strategies. We conclude that the development of extra-somatic weapons throws new light on the evolution of human altruistic and cooperative behaviour, and particularly ‘strong reciprocity’. The notion that distinctively human altruism and cooperation could have been an adaptive trait in a past environment that is no longer evident in the modern world provides a novel addition to theory that seeks to account for this major evolutionary puzzle.

## Introduction

### The puzzle of cooperation and strong reciprocity

Human cooperation and altruism towards non-kin poses two fundamental questions for biology and the behavioural sciences. Firstly, why do genes associated with these behaviours survive when evolutionary theory appears to predict that a ‘cheating’ strategy is fitter and will thus drive such genes to extinction [1], [2]? Secondly, ‘strong reciprocity’ is defined as a propensity to cooperate unconditionally even if this is costly and provides neither present nor future rewards to the co-operator/altruist [3], [4]. Why, therefore, does this behaviour persist as it incurs a cost and/or offers no present or future rewards [3], [4]?

Much current theory suggests that the first question can be resolved by punishment of ‘cheats’ who do not reciprocate altruistic acts i.e. reciprocal altruism [5], [6] and/or by cooperative individuals recognizing each other by reputation and gaining fitness by only associating with other cooperative individuals i.e. indirect reciprocity [7], [8], [9], [10], [11].

‘Strong reciprocity’, however, has been observed in one-off, anonymous encounters under experimental conditions [12] where the reciprocal and reputation effects required by reciprocal altruism or indirect reciprocity theory could not influence the behaviour of participants [4]. Instead, it has been explained by ‘new’ group selection linked to extinction-threatening events [3], [13], to cultural evolution [4], [14], to gene-culture co-evolution [15] and to a combination of the last two factors [16], [17]. Others explain it as a response to conditions of uncertainty in reciprocal relationships [18] or as a maladaptive ‘misfiring’ of evolutionary mechanisms in modern, experimental settings [19].

### Extra-somatic weapons in human evolution

Human cooperation towards non-kin has often been seen as unique in nature [8], [12], [14], [16], [20]. Here, we propose that this very uniqueness might be attributable to unique selection pressures likely to have been present in human evolution itself. The invention of tools and weapons is generally seen as being an important step in human evolution as it enabled hominins to consume a high protein diet through the hunting of game and/or scavenging of meat [21]. Such weapons should be distinguished from somatic weapons (e.g. prominent canine teeth, pronounced musculature), with only humans employing extra-somatic weapons on a widespread and systematic basis. Here, we explore whether their invention might have led to a cultural niche that could have resulted in the evolution of distinctively human cooperation. It has been claimed that cultural processes, particularly in humans, can lead to the creation of niches that change selection pressures to which individuals are exposed, thereby influencing their evolution [22], [23], [24].

The oldest complete hunting weapons yet discovered have been dated to approximately 400,000 years ago [25], although it is speculated that wooden weapons could have existed up to 1 million years before the present [26]. These wooden hunting spears resemble projectile weapons [25] although there is some question as to whether they would have been too heavy to have been used as true projectiles [27] and may have been used instead as thrusting weapons. Recent analysis has suggested that humans first developed long-range projectile weaponry in Africa 70–90,000 years ago [27]. Nearly every known human population over the past 50,000 years has used projectile weapons (e.g. spears and darts, bows and arrows) [28] and those few groups who lacked them (e.g. Tasmanian Aborigines) are known to be descended from populations that did possess them [28].

It has been suggested that the invention of extra-somatic weapons in human evolution could have had an important impact on relationships within hominin groups [5], [6], [29]. In many species, intraspecific conflict has been observed to be of the ‘limited war’ type in which ritualized tactics or inefficient somatic weapons are used to settle disputes without death or serious injury resulting [30]. However, in the case of early humans, the lethal effectiveness of extra-somatic weapons is likely to have selected against those behavioural adaptations that inhibit intraspecific violence observed in many other species. Specifically, the development of extra-somatic weapons could have resulted in:

- an increased frequency of agonistic encounters as dominant individuals, hitherto reliant on physical size, strength and intimidation alone to gain access to valued resources (e.g. mating with females, hunted meat), would have been more open to challenge [29];
- a greater likelihood that such encounters would have proved lethal for the protagonists due to the effectiveness of the weapons and the speed with which they could have resulted in serious injury or death [29] and;
- a consequently greater symmetry or equalization in power between individuals [5], [6], [29] as it could have proved as easy for a subordinate to kill a dominant as *vice versa*.

In such a cultural niche it is likely that natural selection would have discriminated strongly against individuals whose behaviour provoked an above average level of within-group aggression against them. ‘Cheats’ and non-cooperators might well have experienced involvement in such lethal disputes at a higher frequency than others and thus faced a correspondingly greater risk of injury or death. Providing that the costs of involvement in agonistic encounters, where weapons were employed, exceeded the benefits of ‘cheating’ and non-cooperation then such strategies would have proved maladaptive.

In contrast, cooperators inclined to reduce their fitness in order to help others within their group could as a result have been less likely to have become involved in lethal disputes and might consequently have experienced relatively greater fitness. Providing that the cost of this helping behaviour was less than the selective benefit of a reduced frequency of involvement in lethal fights then cooperation could have proved an adaptive strategy in this cultural niche.

To test this hypothesis, we employed two versions of the evolutionary Iterated Prisoner's Dilemma (IPD) model – one in which weapons use was absent and one in which it was present. We used computer simulation to measure the performance of a range of IPD strategies, both cooperative and non-cooperative, in order to quantify the effects of weapons use on them.

## Methods

### The traditional iterated prisoner's dilemma (IPD) model

The IPD model [31], [32] is a classic formulation of how mutual co-operation can evolve in a world of selfish individuals and its process is well known (see Supporting Information, File S1. The iterated prisoner's dilemma (IPD) model). Each player has a choice of whether to cooperate or defect on each move. If both cooperate each receives three points but if both defect each gets only one point. If one cooperates and the other defects then the former receives no points and the latter five points. On any single move it always pays to defect but cooperation has been found to emerge where the endpoint of the series of interactions between two players is unknown [32]. In modelling the IPD, this uncertainty is reflected in the duration of each interaction being determined by a certain probability or discount parameter (see File S1). Computer tournaments have been used to identify those strategies that perform best, with ‘Tit for Tat’ (TFT) (cooperate on the first move and then copy the opponent's last move) [31] and Pavlov [33] generally found to be most successful.

### The evolutionary IPD model

The evolutionary IPD model [34] extends the principle of the traditional IPD model by reflecting the payoffs received by players in one generation in terms of copies of themselves represented in the next generation. Stochastic universal sampling is used to ensure that players produce offspring in proportion to payoffs received so that those with higher payoffs reproduce at a proportionately higher rate than those with lower payoffs. In so far as payoffs reflect fitness the evolutionary IPD model can be seen to mimic natural selection, although recombination and mutation are not simulated.

In our evolutionary IPD model a population of 40 players compete against each other on a round robin basis. We chose 25 widely recognized IPD strategies taken from the scientific literature [34], of which 14 have been classified as cooperative and 11 as non-cooperative [35] (see Supporting Information, File S2: IPD strategies employed).

In each round (see Supporting Information, File S3: Definitions of terms) eight strategies were chosen at random with five players initially adopting a particular strategy. To ensure that each strategy was simulated a sufficient number of times to remove the effects of chance we repeated each round 100 times per generation and averaged the payoffs. Strategies were chosen randomly so that, for example in the initial round, there was an 8/25 = 0.32 probability of any one strategy being chosen. With 100 rounds in that game, each strategy was therefore run an average of 100*0.32 = 32 times. As strategies were subsequently eliminated, this probability was adjusted throughout the competition. With stochastic universal sampling used to choose players for the next generation on the basis of payoffs received in the previous one, the game was repeated for 100 generations, showing how each of the 25 strategies increased, decreased or were eliminated over the duration of the competition.

The performance of each strategy was measured by the number of times each survived for the full 100 generations. As long as a single player adopting a particular strategy was present at the end then that strategy was deemed to have survived. Survival time is seen as providing a comprehensive index of the performance of IPD strategies [35] and in this context offers a measure of the relative fitness of individuals adopting each strategy.

### The ‘weapons use’ IPD model

The ‘weapons use’ IPD model we designed for this study was based on the evolutionary IPD model but we also set out to simulate the effects of extra-somatic weapons on the fitness of players. Our model works on the basis that a defection is, in effect, a refusal to cooperate and an attempt to exploit the other player. It therefore assumes that the more defections there are between players, the greater will be the chance of a dispute occurring between them. It thus captures a key aspect of the real world – that, in relationships with others, ‘cheats’ and uncooperative individuals (i.e. those inclined to defect) are more likely to be involved in disputes than ‘nice’ individuals who are more inclined to cooperate.

When a player accumulated 200 defections as a result of moves both by the player and the player's opponents a dispute was deemed to occur. To simulate an environment of equalized power, each player had the same probability of being eliminated in each dispute (i.e. *p* = 0.05). If either or both were eliminated they took no further part in the competition. If they were not, the process continued until a further 200 defections were accumulated and another dispute was deemed to occur.

Thus, as well as stochastic universal sampling reflecting the payoffs of players who adopt a particular strategy, our model quite separately reflected the effects on fitness of elimination as a result of disputes. In the ‘weapons use’ IPD model, therefore, the performance of each strategy was a measure of both decisions on whether to cooperate or defect and also the effects of the number of defections leading to disputes.

Finally, in all simulations we contrasted the performance of strategies under the evolutionary IPD model (i.e. without weapons use) with that under the ‘weapons use’ IPD model to quantify the impact of weapons use. We were thus able to simulate the relative impact of weapons use on the fitness of individuals adopting each strategy in this environment.

### Other points

Our population of 40 players lies within the typical size range of 15–50 observed in modern hunter/gatherer groups [36]. Nevertheless we are aware that there is mobility between hunter/gatherer groups [37] and that therefore a typical hunter/gatherer would interact with more than 40 individuals over a lifetime. However, we make the simplifying assumption that transfers in and out of groups would have had a broadly neutral effect on the frequency with which each player would encounter the 25 IPD strategies modelled. This point is reflected in the re-running of each round 100 times per generation with different combinations of strategies being encountered in each round.

The ‘weapons use’ IPD model has three key parameters (i.e. average number of interactions with other players in a generation, number of defections before a dispute occurs, probability of being eliminated as a result of a dispute) that, taken together, determine the fitness of each player. The values chosen, however, were not arbitrary but based on a population consisting entirely of players who always defect (AllD) and who are thus at the extreme end of the continuum of behaviour being examined. Based on the values selected, in an AllD population there is a probability of *p* = 0.50 of being eliminated in an average lifetime (see Supporting Information, File S4: The parameters and values used). These values therefore provide a clear benchmark against which the performance of the 25 IPD strategies was calibrated.

## Results

**Figure 1:**
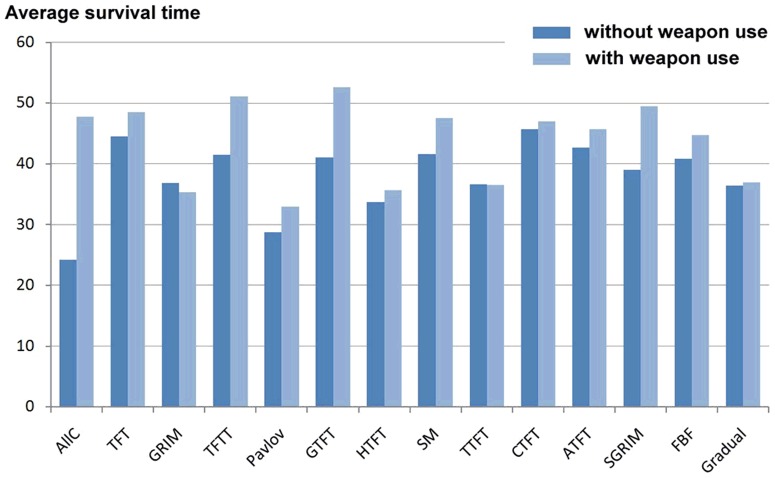
Cooperative strategies: average number of generations survived out of 100 generations (without and with weapons use).

**Figure 2:**
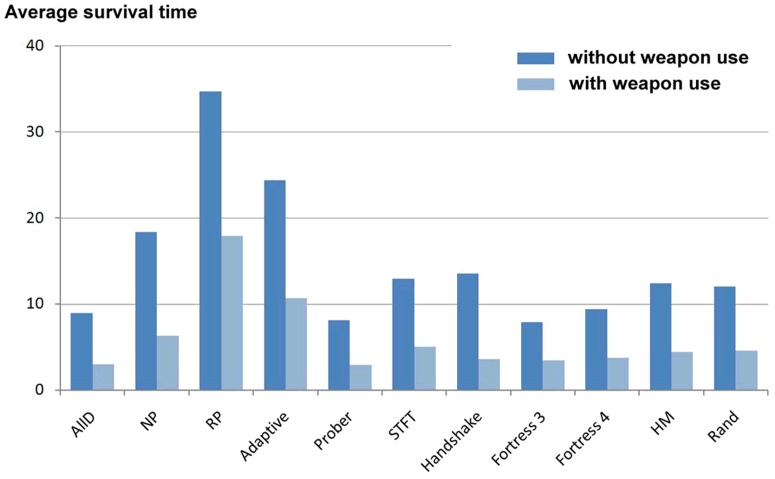
Non-cooperative strategies: average number of generations survived out of 100 generations (without and with weapons use).

The performance of the cooperative and non-cooperative strategies in our sample is illustrated in Figures 1 and 2 respectively. We found that all cooperative strategies survived, on average, an additional 5.6 generations under simulated weapons use (see Table 1), a significant variation (paired samples t-test: *t*_13_ = 3.22; *p* = 0.003 one-tailed). In contrast, we found that non-cooperative strategies survived, on average, 8.8 fewer generations under simulated weapons use (see Table 2), which was also a significant variation (paired samples t-test: *t*_10_ = 7.47; *p* = 0.00001 one-tailed).

**Table 1:** Cooperative strategies: average survival time in 100 evolutionary games (for details of strategies see S 2: IPD strategies employed).

Strategy	Without weapon use (generations) (*p* = 0.00)	With weapon use (generations) (*p* = 0.05)	Change (generations)
AllC	24.26	47.72	23.46
TFT	44.56	48.46	3.90
GRIM	36.84	35.29	−1.55
TFTT	41.44	51.05	9.61
Pavlov	28.75	32.96	4.21
GTFT	41.01	52.66	11.65
HTFT	33.76	35.61	1.85
SM	41.62	47.51	5.89
TTFT	36.62	36.54	−0.08
CTFT	45.65	47.05	1.40
ATFT	42.72	45.72	3.00
SGRIM	38.96	49.53	10.57
FBF	40.84	44.74	3.90
Gradual	36.36	36.94	0.58
**Total**	500.03	577.84	78.39

Note: Average change in performance for all strategies is 78.39/14 = 5.6 generations. Average change in performance of all strategies other than ‘Always Cooperate’ is 54.93/13 = 4.2 generations.

**Table 2:** Non-cooperative strategies: average survival time in 100 evolutionary games (for details of strategies see S 2: IPD strategies employed).

Strategy	Without weapon use (generations) (*p* = 0.00)	With weapon use (generations) (*p* = 0.05)	Change (generations)
AllD	8.92	3.01	−5.91
NP	18.37	6.33	−12.04
RP	34.72	17.93	−16.79
Adaptive	24.41	10.67	−13.74
Prober	8.10	2.93	−5.17
STFT	12.97	5.02	−7.95
Handshake	13.55	3.58	−9.97
Fortress 3	7.90	3.48	−4.42
Fortress 4	9.41	3.75	−5.66
HM	12.43	4.42	−8.01
Rand	12.03	4.57	−7.46
**Total**	162.81	65.69	−97.12

Note: Average change in performance is −97.12/11 = −8.8.

We were surprised at the success of one strategy in particular - ‘Always Cooperate’ (AllC) - in our simulations. When we contrasted the performance of AllC with all other cooperative strategies we found it survived an additional 23.5 generations under simulated weapons use as opposed to an average of only an additional 4.2 generations for all other cooperative strategies (see Table 1). This demonstrated a very significant improvement in performance (one-sample t-test: *t*_11_ = 16.03; *p* = 5.7*1^−9^ one-tailed) for AllC over all other cooperative strategies in this cultural niche.

**Figure 3:**
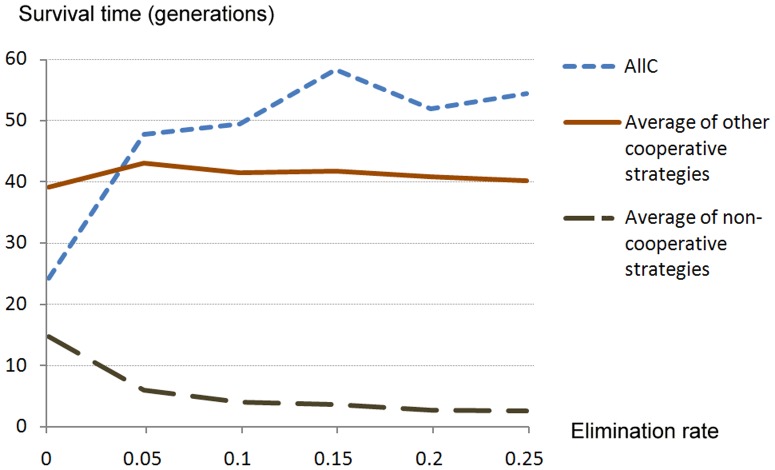
Effects of variation in the elimination rate on: ‘Always Cooperate’ (AllC), the average for other cooperative strategies and the average for all non-cooperative strategies.

Finally, throughout our simulations we have assumed an elimination rate of *p* = 0.05. To explore whether variation in the negative impact of weapons use on fitness might produce different trends we adjusted the elimination rate by stages from *p* = 0.00 (without weapons use) to *p* = 0.25 (or five times the rate used in our original simulation). To simplify illustration of the key patterns we averaged the performance of non-cooperative strategies and contrasted that of AllC with the average for other cooperative strategies (see Figure 3). We found that for non-cooperative strategies and for all cooperative strategies other than AllC the influence of weapons use was most marked between *p* = 0.00 and *p* = 0.05 but thereafter the effect tailed off. In contrast, the performance of AllC generally continued to improve above *p* = 0.05 rather than tailing off. In response to simulated weapons use, AllC improved from being the least successful cooperative strategy at *p* = 0.00 to being the most successful at *p* = 0.15 and *p* = 0.25 (see Table 3).

**Table 3:** Cooperative strategies: effects of variation in elimination rate on survival rate (for details of strategies see S2: IPD strategies employed).

Strategy	Without weapon use (p = 0.00)	With weapon use (varying elimination rates expressed by the value of *p*)				
		0.05	0.10	0.15	0.20	0.25
AllC	24.26	47.72	49.49	58.39	51.90	54.46
TFT	44.56	48.46	45.71	49.69	42.22	43.95
GRIM	36.84	35.29	28.93	31.92	30.68	29.40
TFTT	41.44	51.05	47.05	49.76	53.73	49.41
Pavlov	28.75	32.96	35.96	35.37	35.43	36.65
GTFT	41.01	52.66	53.15	52.25	51.05	51.36
HTFT	33.76	35.61	32.30	28.68	27.92	28.24
SM	41.62	47.51	41.67	41.94	39.20	40.71
TTFT	36.62	36.54	35.94	32.96	33.70	37.95
CTFT	45.65	47.05	41.37	45.41	44.54	40.14
ATFT	42.72	45.72	41.25	44.49	41.18	41.35
SGRIM	38.96	49.53	52.51	48.47	51.35	49.82
FBF	40.84	44.74	48.12	48.59	47.62	42.26
Gradual	36.36	36.94	34.79	33.35	32.66	31.69

## Discussion

Under simulated weapons use, the performance of cooperative strategies improved significantly compared with an environment where weapons use was absent. In contrast, the performance of non-cooperative strategies declined significantly under simulated weapons use compared with an environment where this effect was not modelled. Our hypothesis was therefore supported. The higher incidence of disputes encountered between non-cooperative players and the adverse effect this had on individual fitness appears to explain the patterns found, despite the usual payoffs from the IPD model.

We believe that these findings have important implications for understanding how distinctively human cooperation might have evolved. The traditional IPD model shows how cooperation can emerge in a world of selfish individuals but our model demonstrates that, in an environment of widespread weapons use, this tendency could have been boosted to a substantially greater degree. The invention of extra-somatic weapons has been rightly recognized as an important step in human evolution but this is the first time, as far as we are aware, that its likely indirect impact on human cooperation has been modelled. We therefore consider that these findings provide an important new perspective in helping us to better understand the evolution of human cooperation.

As discussed above, the traditional IPD model and reciprocity theory have difficulty accounting for the evolutionary puzzle of ‘strong reciprocity’ and other forms of the costly punishment of ‘cheats’. The unexpected success of the ‘Always Cooperate’ (AllC) strategy in our simulations, we believe, has important implications for resolving this puzzle. With the traditional IPD model, AllC is easily exploited and is thus relatively unsuccessful [32]. Under the evolutionary IPD model (i.e. without simulated weapons use), AllC proved the least successful of all cooperative strategies (see Table 1). However, despite the lower payoffs that it usually receives and the additional burden of fighting defectors with weapons, an AllC strategy flourished under the ‘weapons use’ IDP model. Its performance improved substantially more than that of all other cooperative strategies in an environment of simulated weapons use (see Table 1) and, from being the poorest performing cooperative strategy in the absence of weapons use, it became the best performing when the negative effect of weapons use was increased to *p* = 0.15 and *p* = 0.25 (see Table 3).

We suggest that the unexpected success of an AllC strategy has important implications for understanding the evolution of ‘strong reciprocity’. The two distinguishing features of ‘strong reciprocity’ are that (i) it is unconditionally cooperative and (ii) it is prepared to inflict costly punishment on ‘cheats’ [3], [4]. Under the ‘weapons use’ evolutionary IPD model, an AllC strategy (i) by definition, cooperates unconditionally and (ii) becomes involved in disputes with defecting strategies that involve an equal probability of elimination from the game. An AllC strategy is therefore equivalent to one of ‘strong reciprocity’ in this cultural niche. The fact that, among all the strategies simulated, AllC responded best to an environment where lethal weapons use was common (see Figure 3 and Table 3) thus provides a new explanation for the evolutionary puzzle of ‘strong reciprocity’.

Our model does not allow players to avoid costly disputes and thus opt out of their adverse effects on individual fitness as this reflects a likely key feature of the development of extra-somatic weapons in human evolution. For, in the resulting environment or cultural niche, it would scarcely have been possible to ‘disinvent’ such weapons or opt out of their use. Attempted invasion of a population of weapons users by those who avoided the use of weapons, and the costs involved, is likely to have resulted in the death of such individuals at the hands of those in possession of superior weapons technology. It would not therefore have been possible to ‘cheat’ by adopting a less costly strategy that avoided weapons use as doing so would have been likely to have resulted in extinction.

One argument that could be raised against our hypothesis is that the development of weapons would have led to variation in the skills needed for their use and would not therefore have resulted in the equalization of power within hominin social groups assumed by our model. We agree that it is likely that differences in skill in weapons use would have emerged in this cultural niche, as suggested by the variation in hunting skills observed in modern hunter/gatherer groups [38]. But this point need not invalidate our hypothesis. The very swiftness and effectiveness with which such weapons could have been employed would have made it relatively easy for any individual to be killed [29]. No one, we suggest, however skilful in the use of weapons, would have been immune to the risk of serious injury or death if they defected too often in interactions with other group members.

Our view is supported by the egalitarian ethos observed in modern hunter/gatherer societies and the extraordinary extent to which such societies go to suppress potential causes of violence [29], [39], [40]. It is also supported by a study of the! Kung where 22 cases of homicide that occurred prior to the introduction of effective legal sanctions by outside civil authorities were examined [39]. Many of these homicides appeared to have had random causes. However, four of these cases of homicide were interpreted as being sanctioned by the community against individuals with a propensity for disruption and violence [39] (i.e. those likely to have consistently defected against other group members). We therefore conclude that it is likely that selection in this cultural niche would have acted primarily against poor co-operators rather than those less skilled in weapons use.

The most important contribution that this study makes, however, is to suggest a novel alternative to explanations of altruism and cooperation based on reciprocal altruism [5] and indirect reciprocity [7], [8]. If the patterns discovered in these simulations were to have been reflected in human evolution, then genes associated with cooperation, altruism and ‘strong reciprocity’ are likely to have increased in frequency in ancestral populations. These selection pressures might even have favoured genes linked to closely allied behaviours and emotions such as prosociality, reciprocity, a sense of ‘fairness’, guilt and moralistic aggression. If so, then genes associated with distinctively human cooperation towards non-kin would come to be expressed in modern populations, like those of any other adaptive behavioural trait, given appropriate stimuli in contemporary environments.

What is being suggested here, therefore, is that distinctively human cooperative and altruistic behaviour evolved because it was an adaptive behavioural trait like any other - but in a cultural niche that no longer exists in the contemporary world (in this case, due to the development of civil societies and modern legal systems suppressing widespread weapons use). Another example of a past environment, no longer evident in the modern world, which might have favoured genes associated with human cooperation and altruism, has been examined elsewhere [41].

What could be called the ‘altruism as an adaptive trait in past environments’ hypothesis can be seen to offer a relatively parsimonious account of this major evolutionary puzzle. It avoids the complex and costly monitoring and punishment of the behaviour of others required by reciprocity theory [9], [42], [43], [44], [45], [46], [47], [48], [49] because the key determinant of altruistic behaviour would simply be the frequency of genes associated with this behaviour across the whole population over evolutionary time. It also avoids the need for repeated interactions [5] and reputation effects [7], [8] required by reciprocity theory. This, of course, has a special relevance to the evidence for ‘strong reciprocity’ described at the start of this article i.e. the experiment that demonstrated altruistic behaviour can persist in the form of altruistic punishment of cheats in one-off, anonymous encounters where reciprocity and reputation effects are not possible [12]. The ‘altruism as an adaptive trait in past environments’ hypothesis is therefore capable of resolving this puzzle by demonstrating that ‘strong reciprocity’ could have been a successful adaptation to a past cultural niche, thus requiring no other mechanism for it to be expressed in modern populations.

The hypothesis tested in this study is based on the intuitively simple notion that the invention of extra-somatic weapons would have made competition far more costly to individual fitness. Intuition, however, is not the same as rigorous testing of a hypothesis. What is much less clear or predictable is how the range of widely contrasting and often complex IPD strategies simulated in this study would perform in a cultural niche where such a condition is commonplace. This point is amply illustrated in the unexpected success of the AllC strategy and the implications which that success has for understanding the origins of human altruism and cooperative behaviour. We consider that the resulting patterns found in this study provide an important new insight into the two major evolutionary puzzles outlined in the Introduction. The challenge now is to test this novel hypothesis by using other methodologies (e.g. a population genetic model) to establish whether the same effects are replicated.

## Supporting Information

File S1 **The iterated prisoner's dilemma (IPD) model.**
(DOCX)

File S2 **IPD strategies employed.**
(DOCX)

File S3 **Definitions of terms.**
(DOCX)

File S4 **The parameters and values used.**
(DOCX)
