# Supplementary material for: The Effects of Extra-Somatic Weapons on the Evolution of Human Cooperation towards Non-Kin
Source: PLoS One. 2014 May 5;9(5):e95742. doi: 10.1371/journal.pone.0095742 (PMC4010415; doi:10.1371/journal.pone.0095742)
Supplement: File S1 — The iterated prisoner's dilemma (IPD) model. (DOCX) [file pone.0095742.s001.docx]

**Supporting information file S1: The iterated prisoner’s dilemma (IPD) model**

The IPD model is an abstract formulation of how mutual co-operation can evolve in the world of selfish individuals [1]. It is based on an imaginary scenario in which the police interrogate two hypothetical prisoners in separate rooms. If both refuse to blame the other for the crime of which they are accused each receives only a modest sentence, as the police evidence against them is not compelling. But if one blames the other prisoner and that prisoner continues to keep quiet the confessing prisoner is let off scot-free and the silent prisoner receives the maximum sentence.

This imaginary scenario, however, captures in a simplified form the uncertain balance of advantage and disadvantage between cooperation and non-cooperation in encounters between individuals - and the universal dilemma to which it gives rise. In any single interaction between the two prisoners (or players) it pays both to defect. However, where interactions between the two players are repeated over an indefinite period of time it has been found that cooperation emerges, thus helping to resolve the puzzle of how cooperation can develop in the world of selfish individuals predicted by evolutionary theory.

In terms of the scores or payoffs from the IPD model, where both players cooperate each receives a Reward *R* = 3 points. Where both defect each receives a Punishment *P* = 1 points. Where one cooperates and the other defects, the co-operator receives the Sucker’s Payoff *S* = 0 points while the defector gets the Temptation to defect *T* = 5 points. These payoffs, which are arranged so that *T* > *R* > *P* > *S*, are summarized below:

| Column Player | | | |
| --- | --- | --- | --- |
| Row  Player |  | Cooperate | Defect |
|  | Cooperate | *R = 3, R = 3*  Rewards for mutual cooperation | *S = 0, T = 5*  Sucker’s payoff and temptation to defect |
|  | Defect | *T = 5, S = 0*  Temptation to defect and sucker’s payoff | *P = 1, P = 1*  Punishment for mutual defection |

In our models, the duration of an interaction is determined by a probability of p = 0.02 that it will come to an end with each move. This results in the average length of a generation being 50 moves by an individual player or 100 moves by both. A series of IPD strategies have been devised and computer simulation used in tournaments between these strategies to determine which is most likely to result in successful cooperation.

[1] Axelrod R (1984) The evolution of cooperation. London: Penguin Books.
