# Supplementary material for: The Effects of Extra-Somatic Weapons on the Evolution of Human Cooperation towards Non-Kin
Source: PLoS One. 2014 May 5;9(5):e95742. doi: 10.1371/journal.pone.0095742 (PMC4010415; doi:10.1371/journal.pone.0095742)
Supplement: File S2 — IPD strategies employed. (DOCX) [file pone.0095742.s002.docx]

**Supporting information file S2: IPD strategies employed**

We define cooperative strategies as those that will never defect before their opponent defects [1]. Two cooperative strategies will therefore always cooperate with each other in a non-stochastic IPD game. We define non-cooperative strategies as those that, regardless of short-term tactics, will always ultimately seek to maximize payoffs by defecting against an opponent [1]. The 14 cooperative IPD strategies employed are defined as follows:

**Always Cooperate (AllC):** Cooperates on every move.

**Tit for Tat (TFT):** Cooperates on the first move, then copies the opponent’s last move.

**Tit for Two Tats (TFTT):** Cooperates on the first move, and defects only when the opponent defects two times.

**Grudger (GRIM):** Cooperates, until the opponent defects, and thereafter always defects.

**Pavlov:** Cooperates on the first move. If a reward or temptation payoff is received in the last round then repeats last choice, otherwise chooses the opposite choice.

**Generous Tit for Tat (GTFT):** Same as TFT, except that it cooperates with a probability q = 0.33 when the opponent defects.

**Hard Tit for Tat (HTFT):** Cooperates on the first move, and defects if the opponent has defects on any of the previous three moves, else cooperates.

**Soft Majority (SM):** Cooperates on the first move, and cooperates as long as the number of times the opponent has cooperated is greater than or equal to the number of times it has defected, else it defects.

**Two Tits for Tat (TTFT):** same as Tit for Tat except that it defects twice when the opponent defects.

**Contrite Tit for Tat (CTFT):** Same as TFT except that it will occasionally choose cooperate twice when it receives T in order to recover mutual cooperation.

**Adaptive Tit for Tat (ATFT):** An adaptation rate r determines what ATFT chooses [2]. This is computed from the history of moves of the opponent and the opponent’s behaviour.

**Soft Grudger (SGRIM):** Like GRIM except that the opponent is punished with D,D,D,D,C,C.

**Firm But Fair (FBF):** cooperates on the first move, and cooperates except after receiving a sucker payoff.

**Gradual:** Cooperates on the first move, and cooperates as long as the opponent cooperates. After the first defection of the other player, it defects one time and cooperates two times. After the nth defection it reacts with n consecutive defections and then calms down its opponent with two cooperates.

The 11 non-cooperative IPD strategies employed are defined as follows:

**Always Defect (AllD):** Defects on every move.

**Adaptive:** Starts with C,C,C,C,C,C,D,D,D,D,D and then takes choices which have given the best average score recalculated after every move.

**Hard Majority (HM):** Defects on the first move, and defects if the number of defections of the opponent is greater than or equal to the number of times it has cooperated, else cooperates.

**Remorseful Prober (RP):** Like Naïve Prober, but it tries to break the series of mutual defections after defecting.

**Naïve Prober (NP):** Like Tit for Tat, but occasionally defects with a small probability of *p* = 0.05.

**Prober:** Starts with D,C,C and then defects if the opponent has cooperated in the second and third move. Otherwise, it plays TFT.

**Suspicious Tit for Tat (STFT):** Same as TFT, except that it defects on the first move.

**Handshake:** Defects on the first move and cooperates on the second move. If the opponent behaves the same as Handshake does, it always cooperates. Otherwise, it always defects.

**Fortress 3:** Like Handshake, it tries to recognize a kin member by playing D,D,C. If the opponent plays the same sequence of D,D,C it cooperates until the opponent defects. Otherwise, it defects until the opponent defects on two continuous moves, and then it cooperates on the following move.

**Fortress 4:** Same as Fortress3 except that it plays D,D,D,C in recognizing kin members. If the opponent plays the same sequence of DDDC it cooperates until the opponent defects. Otherwise, it defects until the opponent defects on three continuous moves and then cooperates on the following move.

**Random Player (RAND):** Makes only random moves.

[1] Li J, Kendall G (2009) A strategy with novel evolutionary features for the iterated prisoner’s dilemma. Evol Comput 17: 257-274.

[2] Tzafestas E (2000) Toward adaptive cooperative behaviour. Proceedings of the Simulation of Adaptive Behavior Conference, 334-340.
