# Supplementary material for: The Effects of Extra-Somatic Weapons on the Evolution of Human Cooperation towards Non-Kin
Source: PLoS One. 2014 May 5;9(5):e95742. doi: 10.1371/journal.pone.0095742 (PMC4010415; doi:10.1371/journal.pone.0095742)
Supplement: File S3 — Definitions of terms. (DOCX) [file pone.0095742.s003.docx]

**Supporting information file S 3: Definitions of terms**

| **Competition** | A series of simulations over 100 generations |
| --- | --- |
| **Game** | 100 rounds designed to represent the performance of all strategies averaged over one generation |
| **Round** | A simulation in which five players adopting one strategy interact initially with other groups of five players adopting different strategies i.e. with 40/5 = 8 strategies used each round |
| **Population** | The number of players in each round (i.e. 40) |
| **Interaction** | Simultaneous decisions on whether to cooperate or defect by two players, a series of which are brought to an end by the discount parameter |
| **Discount parameter** | A probability of *p* = 0.02 on completion of an interaction between players that future interactions between them will cease |
| **Move** | A decision by an individual player on whether to cooperate or defect as part of an interaction |
