# Supplementary material for: The Effects of Extra-Somatic Weapons on the Evolution of Human Cooperation towards Non-Kin
Source: PLoS One. 2014 May 5;9(5):e95742. doi: 10.1371/journal.pone.0095742 (PMC4010415; doi:10.1371/journal.pone.0095742)
Supplement: File S4 — The parameters and values used. (DOCX) [file pone.0095742.s004.docx]

**Supporting information file S 4: The parameters and values used**

Under the ‘weapons use’ IPD model the performance of each player is reflected not only in the usual payoffs resulting from the evolutionary IPD model but also in the following parameters:

- The average number of moves by a player in a generation. This is based on population size (i.e. 40) and the discount parameter of *p* = 0.02 which determines the average length of interactions between two players;
- The number of defections between two players before a dispute is deemed to occur (i.e. 200 defections);
- The probability of an individual player being eliminated from the game when a dispute occurs (i.e. an elimination rate of *p* = 0.05).

These values are not arbitrary. They are based on what would happen in a population consisting entirely of AllD players and thus represents the extreme end of the spectrum of behaviour being examined here. Their combined effect is designed to ensure that, in a population of AllD players, there is a probability of *p* = 0.50 of being eliminated in an average lifetime. This is calculated below.

A discount parameter of *p* = 0.02 results in a 2 in 100 or 1 in 50 probability of an interaction coming to an end or an average of 50 interactions between two players. Thus an AllD individual would make 50 moves (or in this case, defections) in an average interaction with another AllD individual. But that same individual also interacts with 40 other AllD players resulting in 50 x 40 = 2000 defections per individual in a generation.

The number of defections between 2 players before a dispute is called is 200 interactions (or 200 moves which are, in this case, defections) per individual AllD player. Therefore for a single AllD individual in a population of only AllD players this results in 2000/200 = 10 disputes per individual in a lifetime. But the elimination rate is *p* = 0.05 or 1 in 20. Therefore an AllD player who plays only other AllD players would experience a probability of being eliminated from the game of:

*p* = 10 * 0.05 = 0.50.

This combination of parameters can thus be seen to provide a clear benchmark against which the performance of players adopting all the other IPD strategies employed in this model can be assessed.
